# Researchers' experiences of optimising complex health interventions before full-scale RCTs: results from an exploratory multiple case study

**DOI:** 10.1186/1745-6215-16-S2-O29

**Published:** 2015-11-16

**Authors:** Sara Levati, Suzanne Hagen, Cam Donaldson, Mary Wells

**Affiliations:** 1 NMAHP Research Unit - Glasgow Caledonian University, Glasgow, UK; 2 NMAHP Research Unit - University of Stirling, Stirling, UK; 3 Yunus Centre for Social Business and Health, Glasgow Caledonian University, Glasgow, UK

## Background

Given the current financial constraints upon health services research and the failure of many trials to establish whether the lack of intervention effect is due to sub-optimal intervention design or implementation failure, it is increasingly important to consider pre-trial methods that a) provide leads on how the intervention works, and b) help maximise chances for the intervention to be effective. However, the decision-making processes of, and specific benefits associated with conducting pre-trial optimisation studies are still unclear. This multiple case study was conducted to gain researchers' experiences and views of the process of optimising complex health interventions.

## Methods

This exploratory multiple case study focused on a spectrum of different types of complex interventions. A scoping review conducted in advance informed the design, sampling and methods adopted. A total of 15 cases were explored in-depth, using semi-structured interviews as the main data collection strategy.

## Results

Among the main benefits, optimisation processes allowed researchers to anticipate the potential effects of the intervention and to establish whether to embark on a costly full-scale RCT. However, the usefulness of optimisation may be limited by the assumptions and hypothetical circumstances on which processes are often based. Decisions about which optimisation methods to adopt are influenced by several elements, including the evidence available, the experience of the research team, the target of the intervention (i.e. single individuals or groups), and the mechanisms of action of the intervention.

## Conclusions

The findings will inform the development of optimisation study guidelines for researchers striving to develop effective complex health interventions.

